# A Teaching Reform Practice to Improve Research Literacy of Veterinary Postgraduate Students Based on Evidence-Based Veterinary Medicine

**DOI:** 10.3390/vetsci13030281

**Published:** 2026-03-18

**Authors:** Wanglong Zheng, Penggang Liu, Bin Li, Huiling Zhang, Xinyuan Liu, Tangjie Zhang

**Affiliations:** 1 Institute of Comparative Medicine, College of Veterinary Medicine, Yangzhou University, Yangzhou 225009, China; wanglongzheng@yzu.edu.cn (W.Z.); liupg2018@yzu.edu.cn (P.L.); 008480@yzu.edu.cn (B.L.); 18795961172@163.com (X.L.); 2 Jiangsu Co-Innovation Center for Prevention and Control of Important Animal Infectious Diseases and Zoonoses, Yangzhou 225009, China; 3 Independent Researcher, New York, NY 11355, USA; huz21@pitt.edu

**Keywords:** evidence-based veterinary medicine, veterinary postgraduate education, research capacity building, meta-analysis training, educational training

## Abstract

Despite the importance of researcher literacy in veterinary postgraduate education, conventional training often overlooks the methodologies of evidence synthesis. This study assessed an evidence-based veterinary medicine (EBVM) pathway that integrates structured meta-analysis practice into the curriculum. Veterinary postgraduates were assigned to either an EBVM-intensive training group or a comparison group receiving routine instruction. Pre- and post-intervention assessments using a structured questionnaire revealed that the training group achieved superior proficiency in literature retrieval, critical appraisal, and methodological rigor. Notably, this pedagogical approach yielded nine peer-reviewed meta-analyses between 2021 and 2025, while no comparable output was observed in the comparison group. This evidence suggests that integrating hands-on meta-analysis into EBVM instruction serves as a catalyst, transforming theoretical learning into tangible, high-quality scholarly output.

## Simple Summary

Developing researcher literacy is fundamental to veterinary postgraduate education, yet traditional training often prioritizes laboratory-based research over evidence synthesis. This study evaluates an evidence-based veterinary medicine (EBVM) pathway integrating systematic review and meta-analysis practice. Compared to routine instruction, students in the training group showed superior gains in systematic literature searching, methodological rigor, and scientific writing. Crucially, the training group translated these skills into nine peer-reviewed publications—an output absent in the comparison group. These results indicate that embedding structured evidence synthesis into veterinary curricula may provide a practical approach for linking classroom learning and professional academic productivity.

## 1. Introduction

Evidence-based medicine (EBM) was originally developed in human medicine as an approach to support clinical decision-making through the integration of research evidence, professional expertise, and patient values [1]. Within this context, systematic reviews and meta-analyses have gradually become important tools for summarizing existing evidence and informing clinical practice. Over time, meta-analysis has been widely adopted in both medical research and education [2,3,4]. At the same time, the growing number of meta-analytical studies has prompted concerns about inappropriate use, duplication of research topics, and limited added value in certain cases [5,6,7].

The application of evidence-based principles in veterinary medicine has been comparatively slower [8,9]. Evidence-based veterinary medicine (EBVM), introduced in the early 2000s, was intended to promote more structured and transparent decision-making in veterinary research and practice [10,11]. Although the importance of EBVM is increasingly acknowledged, its implementation in veterinary postgraduate education remains uneven. In many programs, training related to evidence-based methods is still largely theoretical, and opportunities for students to apply these concepts in real research settings are limited [12,13]. As a result, postgraduate students may encounter challenges when independently conducting research, particularly in literature searching, critical appraisal, and academic writing.

Against this background, meta-analysis may serve as a practical entry point for strengthening research training in veterinary postgraduate education. Conducting a meta-analysis requires students to define research questions clearly, carry out systematic literature searches, evaluate study quality, and interpret synthesized results. These tasks reflect essential research skills rather than isolated methodological techniques. In addition, given the relatively small and heterogeneous evidence base in veterinary medicine [14,15], meta-analysis can offer a feasible way for postgraduate students to contribute to the literature while developing core research competencies.

In China, early discussions of EBVM mainly focused on its conceptual development and application prospects, with relatively little emphasis on structured educational training or its outcomes [16,17,18]. More recent exploratory studies have suggested that introducing evidence-based approaches into veterinary postgraduate training may improve students’ research awareness and learning motivation. However, most of these studies relied on descriptive observations and lacked standardized evaluation methods or clearly defined academic outputs [19]. Consequently, evidence demonstrating whether EBVM-oriented training leads to measurable improvements in postgraduate research competence remains limited.

In recent years, China has experienced a rapid increase in the number of published meta-analyses across biomedical disciplines, including veterinary science. While this trend reflects growing research activity, concerns have emerged regarding methodological rigor and potential redundancy, echoing issues previously observed in human medicine. Without structured training in evidence synthesis and critical appraisal, the expansion of meta-analytic publications may risk reduced quality and inefficient use of research resources. Therefore, strengthening formal EBVM training within postgraduate curricula is essential to ensure methodological integrity and responsible research practices.

To address this issue, the present study implemented an EBVM-guided training program centered on hands-on meta-analysis practice for veterinary postgraduate students. By combining systematic instruction with iterative research tasks, this study aimed to examine changes in students’ research competence across multiple dimensions and to assess whether these changes were reflected in tangible academic outputs.

## 2. Materials and Methods

### 2.1. Study Design and Participants

This study employed a quasi-experimental, parallel-group design to evaluate the effects of an EBVM-guided training program on postgraduate researcher literacy. Veterinary graduate students enrolled between 2019 and 2024 at a single veterinary medical institution were included. Participants were allocated to either a training group receiving EBVM-based training or a comparison group following the conventional postgraduate training curriculum. Students were grouped according to the curriculum structure in place at the time of their enrollment. The EBVM-oriented training was implemented as a scheduled curriculum reform and was not choice-based.

To reduce the influence of annual cohort size fluctuations, participant data were aggregated into two-year periods (2019–2020, 2021–2022, and 2023–2024) for analysis. The training group comprised 18–20 students per two-year period, while the comparison group included approximately 20 students per period. Female students accounted for a higher proportion in both groups, consistent with the overall gender distribution of veterinary postgraduate enrollment.

### 2.2. EBVM-Based Training

The training program was designed according to core EBVM principles, including structured research question formulation, systematic literature retrieval, critical appraisal of evidence, and quantitative synthesis of research findings [10,11]. Meta-analysis was selected as the central pedagogical tool to operationalize these principles within authentic research settings.

Students in the training group received stepwise training covering topic selection, database searching, methodological quality assessment, data extraction, statistical synthesis, and interpretation of pooled results. International reporting standards, particularly the PRISMA 2020 guidelines, were explicitly introduced and applied throughout the training process to ensure methodological rigor and transparency [20]. Each student was required to complete at least one full meta-analysis project under faculty supervision, progressing from protocol development to manuscript preparation.

### 2.3. Conventional Training in the Comparison Group

Students in the comparison group followed the standard postgraduate training pathway, which primarily emphasized experimental design, laboratory-based research, and thesis-oriented instruction. Although general literature review skills were introduced, no structured EBVM framework or systematic meta-analysis training was provided.

### 2.4. Assessment of Researcher Literacy

Researcher literacy was assessed using a structured questionnaire adapted from established evidence-based practice and research competency frameworks [12]. The instrument evaluated four core dimensions: (1) literature retrieval and information management, (2) critical appraisal and evidence evaluation, (3) research design and analytical reasoning, and (4) academic writing and scholarly communication.

The questionnaire was administered before and after the training period. Internal consistency was assessed using Cronbach’s alpha, with values exceeding 0.80 across all dimensions, indicating good reliability. Construct validity was supported through expert review and consistency with previously reported framework structures. The complete questionnaire items and detailed scoring procedures are provided in Supplementary File S1 to enhance transparency and allow for replication in other educational settings.

### 2.5. Academic Output Assessment

To complement self-reported literacy measures, objective academic output was also evaluated. Peer-reviewed meta-analyses published by students in the training group during and after the training period were recorded. A total of nine meta-analysis articles published between 2021 and 2025 were identified and verified. These publications served as tangible indicators of the translation of researcher literacy into scholarly productivity.

### 2.6. Ethical Considerations

This study was reviewed and approved by the institutional ethics committee of the participating institution. The participants were postgraduate students enrolled in a routine curriculum-based training program, and the EBVM-oriented training was implemented as part of regular educational activities rather than as a separate experimental intervention. All data were collected and analyzed retrospectively for educational evaluation purposes and were fully anonymized prior to analysis. According to institutional policy, individual written informed consent was not required.

### 2.7. Statistical Analysis

Descriptive statistics were calculated for demographic variables and researcher literacy scores. Changes in literacy scores within and between groups were compared using the appropriate parametric or non-parametric tests based on data distribution. Statistical significance was set at *p* < 0.05. All analyses were conducted using standard statistical software.

## 3. Results

### 3.1. Participant Characteristics

A total of 116 postgraduate students participated in the study, with 56 in the training group and 60 in the comparison group, aggregated across three two-year periods (2019–2020, 2021–2022, 2023–2024). Female students accounted for more than half in both groups (training: 71%; comparison: 70%). Baseline characteristics, including age, prior research experience, and field of study, were similar between groups (Table 1), indicating comparability before the training.

### 3.2. Effect of EBVM Training on Researcher Literacy

Pre-training assessment showed no statistically significant difference in overall researcher literacy scores between the training and comparison groups (mean ± SD: 68.4 ± 6.2 vs. 67.9 ± 5.8, *p* = 0.68). Following the training, the training group showed a statistically significant improvement in overall researcher literacy (pre: 68.4 ± 6.2; post: 83.7 ± 5.4; *p* < 0.001), whereas the comparison group showed minimal change (pre: 67.9 ± 5.8; post: 70.2 ± 6.0; *p* = 0.12). Improvements were observed across all four dimensions, with the largest gains in critical appraisal (mean increase: 18%) and literature retrieval (mean increase: 15%) (Table 2).

The training emphasized systematic review methodology, data extraction, and manuscript preparation through structured meta-analysis practice. No additional writing-specific training was provided beyond the embedded curriculum activities.

### 3.3. Conceptual Framework

**Figure 1:**
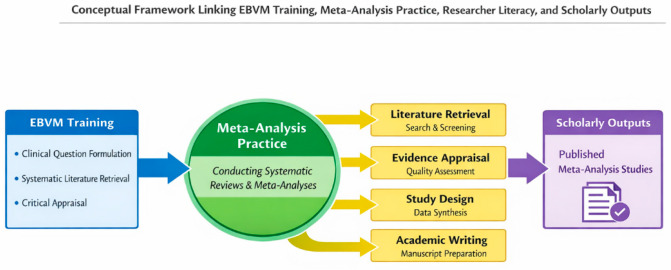
Conceptual framework of EBVM training and its impact on researcher literacy. EBVM components foster skill development, leading to improved literacy scores and peer-reviewed publications.

Figure 1 illustrates the framework of how structured EBVM instruction and hands-on meta-analysis projects enhance postgraduate students’ research competencies across multiple dimensions, leading to measurable improvements in academic output and scholarly productivity.

### 3.4. Academic Output: Meta-Analysis Publications as an Indicator of Researcher Literacy

During and after the implementation of the evidence-based veterinary medicine (EBVM)-oriented training program, students in the training group collectively published nine peer-reviewed meta-analysis articles between 2021 and 2025 (Table 3) [21,22,23,24,25,26,27,28,29]. These publications represent a concrete and verifiable outcome of the educational training, reflecting the transformation of research literacy from abstract competencies into measurable scholarly performance.

Importantly, conducting a meta-analysis requires a coordinated set of advanced research skills, including systematic literature retrieval, critical appraisal of study quality, standardized data extraction, statistical synthesis, and transparent reporting. The successful completion and publication of these studies therefore indicates not only topic-specific knowledge acquisition but also the development of integrated methodological competence. In contrast, no comparable publications were produced by students in the comparison group during the same period, suggesting that the observed academic outputs were closely associated with participation in the EBVM training pathway rather than routine postgraduate training alone.

The thematic diversity of the published meta-analyses—covering viral diseases in poultry, companion animal infectious diseases, parasitic infections in captive wildlife, and nutritional trainings in livestock—further suggests that the acquired research competencies were transferable across different domains of veterinary science. Moreover, all nine studies adhered to established reporting standards for systematic reviews and meta-analyses, indicating improved awareness of research transparency and methodological rigor.

Taken together, these publication outcomes provide strong supplementary evidence that EBVM-oriented training, particularly when combined with hands-on meta-analysis practice, can effectively enhance postgraduate researcher literacy and facilitate the translation of educational inputs into high-quality scientific outputs.

## 4. Discussion

### 4.1. Researcher Literacy in Veterinary Postgraduate Education: Current Context

Researcher literacy has increasingly been recognized as a core competency in postgraduate education, encompassing not only technical research skills but also the ability to identify meaningful questions, critically appraise evidence, and communicate findings transparently. In human medicine, evidence-based medicine (EBM) and meta-analysis have long been integrated into postgraduate training frameworks [30,31,32]; however, concerns have also been raised regarding the overproduction and methodological misuse of meta-analyses [33,34,35], which may dilute scientific value when not accompanied by adequate training and critical appraisal skills [5,36]. Previous analyses have suggested that the rapid proliferation of meta-analyses in China may, in part, be driven by academic evaluation systems that emphasize publication quantity. This has led to increasing concerns about redundancy, inconsistent methodological quality, and the potential dilution of high-quality evidence in the medical literature [37,38,39]. In contrast, the systematic application of evidence-based veterinary medicine (EBVM) in postgraduate education remains relatively recent [40,41]. Previous surveys have shown that although veterinarians generally acknowledge the importance of EBVM, their actual proficiency in evidence appraisal and synthesis is often limited [13,42]. In China, early discussions on evidence-based veterinary medicine (EBVM) education emphasized its potential benefits, while also identifying structural barriers, including limited formal training in research methodology and insufficient exposure to systematic reviews and meta-analyses during postgraduate education [18,19]. These observations underscore the need for structured educational pathways that integrate EBVM principles with practical research training.

### 4.2. Effectiveness of EBVM-Oriented Training on Researcher Literacy

The present study suggests that an EBVM-oriented training pathway can lead to significant improvements in overall researcher literacy among veterinary postgraduate students. Compared with routine training, the training group showed greater post-training gains across multiple dimensions, including literature retrieval, critical appraisal, methodological understanding, and scientific writing. These findings align with prior reports suggesting that active engagement with EBVM tools enhances analytical thinking and research competence beyond passive knowledge acquisition [10,11].

Importantly, the observed improvements were not limited to self-reported perceptions but were reflected in structured questionnaire scores with acceptable reliability and validity. The female predominance observed in this cohort is consistent with the gender distribution commonly reported in veterinary postgraduate education, reflecting the broader feminization trend of the veterinary profession [43].

### 4.3. Meta-Analysis Practice as a High-Level Integrative Training Tool

A key feature distinguishing the training pathway was the incorporation of hands-on meta-analysis practice. Meta-analysis is inherently integrative, requiring students to synthesize skills in systematic searching, study selection, bias assessment, data extraction, statistical pooling, and standardized reporting [20]. Unlike single experimental studies, meta-analyses demand sustained engagement with the literature and repeated methodological decision-making, making them particularly suitable for researcher literacy training when appropriately supervised.

While concerns have been raised in human medicine regarding the proliferation of redundant or low-quality meta-analyses, the veterinary field remains in an earlier stage of evidence-based practice development, where carefully guided training in systematic reviews and meta-analytic methods could further enhance research skills and clinical decision-making [44,45,46,47]. When embedded within an EBVM framework, meta-analysis serves not only as a research output but also as a structured learning process that reinforces evidence-based reasoning [15,48].

### 4.4. Academic Output as Evidence of Translational Research Competence

The publication of nine peer-reviewed meta-analysis articles by the training group between 2021 and 2025 provides concrete evidence that improvements in researcher literacy translated into sustained scholarly productivity (Table 3). These publications covered diverse veterinary domains, including poultry viral diseases, companion animal infectious diseases, parasitic infections in captive wildlife, and nutritional training in livestock production. Such thematic breadth suggests that the acquired competencies were transferable across subdisciplines, rather than restricted to a narrow research niche.

Notably, no comparable meta-analysis publications were produced by the comparison group during the same period. Although a proportion of students had prior research experience before enrollment, structured EBVM training appeared to enhance researcher literacy beyond baseline research exposure. Although academic output can be influenced by multiple factors, the temporal consistency observed between EBVM-oriented training and subsequent publication output in this study indicates a potential causal link, consistent with evidence reported in previous studies in both veterinary [41,42,43,44,45,46,47,48,49] and human medical education [50,51]. Moreover, adherence of these studies to internationally recognized reporting standards further indicates improved awareness of research rigor and transparency.

### 4.5. Educational Implications and Limitations

From an educational perspective, these findings suggest that EBVM-oriented training pathways, particularly those incorporating supervised meta-analysis practice, may offer a scalable and methodologically robust approach to improving postgraduate researcher literacy in veterinary medicine. Such pathways may be particularly valuable in settings where experimental infrastructure and research investment are constrained, but access to published evidence and secondary data sources is relatively abundant [52,53].

Despite the promising findings, several limitations warrant consideration. The relatively small cohort and single-center, quasi-experimental framework inherently restrict the generalizability of our results and may introduce selection bias. The modest cohort size precluded subgroup analyses (e.g., by gender), and systematic long-term follow-up data were not collected. In addition, the researcher literacy instrument relied on self-reported responses and was adapted from existing frameworks rather than validated in veterinary postgraduate populations; thus, improvements may partly reflect increased confidence and skill acquisition. It is also important to note that this study prioritized immediate academic productivity; consequently, the long-term retention of these competencies remains to be established. To build upon these preliminary results, future research should employ multi-center, randomized designs with longitudinal tracking to evaluate the enduring impact of this pedagogical model.

## 5. Conclusions

An evidence-based veterinary medicine (EBVM)–oriented training pathway, enriched by structured meta-analysis practice, provides a framework for enhancing researcher literacy among veterinary postgraduates. This approach extends beyond traditional theoretical instruction and may facilitate the translation of pedagogical gains into measurable scholarly output. These findings suggest that integrating EBVM principles with meta-analysis practice represents a promising, exploratory strategy for strengthening postgraduate veterinary curricula, although longer-term evaluation is warranted.

## Figures and Tables

**Table 1:** Demographic and baseline characteristics of participants by group and period.

Period	Group	N	Female, n (%)	Age, Mean ± SD	Prior Research Experience, n (%)
2019–2020	Training	18	13(72)	23.8 ± 1.2	6(33)
	Comparison	20	14(70)	23.5 ± 1.1	7(35)
2021–2022	Training	20	14(70)	24.0 ± 1.3	8(40)
	Comparison	20	14(70)	23.7 ± 1.0	7(35)
2023–2024	Training	18	14(72)	24.2 ± 1.1	6(33)
	Comparison	20	14(70)	24.0 ± 1.2	7(35)

Percentages are rounded to the nearest whole number. Prior research experience refers to participation in independent research activities before enrollment. No significant differences in baseline characteristics were observed between the training and comparison groups (*p* > 0.05).

**Table 2:** Researcher literacy scores before and after the training by group.

Dimension	Training Pre	Training Post	Cohen’s d (95% CI)	Comparison Pre	Comparison Post	Cohen’s d (95% CI)
Literature Retrieval	17.2 ± 2.1	19.8 ± 1.5	2.43 (2.00–2.86)	17.0 ± 2.0	17.5 ± 1.9	0.40 (0.05–0.75)
Critical Appraisal	16.5 ± 2.3	19.5 ± 1.6	2.68 (2.25–3.10)	16.8 ± 2.1	17.0 ± 1.8	0.40 (0.05–0.75)
Research Design & Analysis	18.3 ± 2.0	21.0 ± 1.5	2.77 (2.35–3.19)	18.1 ± 1.9	18.5 ± 1.8	0.37 (0.02–0.72)
Academic Writing	16.4 ± 2.1	23.4 ± 1.8	3.04 (2.60–3.48)	16.0 ± 2.0	17.2 ± 1.9	0.23 (–0.10–0.56)
Total Score	68.4 ± 6.2	83.7 ± 5.4	3.79 (3.30–4.28)	67.9 ± 5.8	70.2 ± 6.0	0.50 (0.15–0.85)

Values are presented as mean ± SD; Cohen’s d (95% CI) indicated effect size for Pre→Post changes. All post-training scores in the training group were significantly higher than the comparison group (*p* < 0.001 for total score and all dimensions).

**Table 3:** Meta-analysis publications by training group students (2021–2025).

Year	First Author	Title	Journal	IF
2021	Zhou S. [21]	Effects of different selenium sources on sow reproductive performance and piglet development: meta-analysis	J Anim Feed Sci	1.5
2021	Zhou S. [22]	Seroprevalence of Toxoplasma gondii in cats in mainland China (2016–2020)	J Vet Sci	1.6
2022	Zhou S. [23]	Duck hepatitis A virus prevalence in mainland China (2009–2021): systematic review and meta-analysis	Prev Vet Med	2.7
2023	Ye L. [24]	A meta-analysis for vaccine protection rate of duck hepatitis A virus in mainland China (2009–2021)	BMC Vet Res	2.6
2024	Hu T. [25]	Prevalence and risk factors associated with feline infectious peritonitis in mainland China (2008–2023)	Animals	2.7
2024	Hong X. [26]	Meta-analysis for prevalence of infectious laryngotracheitis in chickens in mainland China (1981–2022)	BMC Vet Res	2.6
2025	Zhang X. [27]	The occurrence and meta-analysis of intestinal parasitic infections among captive wild mammals in mainland China	Vet Sci	2.3
2025	Zhang X. [28]	Global prevalence of Psittacine Beak and Feather Disease Virus infection and associated risk factors: meta-analysis	Animals	2.7
2025	Zhou H. [29]	Prevalence and Risk Factors of Mycoplasma Hyopneumoniae in Swine Farms, Mainland China, 2003–2024: A Meta-Analysis	Vet Sci	2.3

## Data Availability

The original contributions presented in this study are included in the article and Supplementary Material. Further inquiries can be directed to the corresponding author.

## Supplementary Materials

The following supporting information can be downloaded at: https://www.mdpi.com/article/10.3390/vetsci13030281/s1, File S1: Researcher literacy questionnaire and scoring framework.
