# Supplementary material for: A Teaching Reform Practice to Improve Research Literacy of Veterinary Postgraduate Students Based on Evidence-Based Veterinary Medicine
Source: Vet Sci. 2026 Mar 18;13(3):281. doi: 10.3390/vetsci13030281 (PMC13030425; doi:10.3390/vetsci13030281)
Supplement: Supplementary file 1 [file vetsci-13-00281-s001.zip › vetsci-4156458-supplementary.pdf]

Supplementary File S1

Researcher Literacy Questionnaire and Scoring Framework

1. Instrument Structure

The Researcher Literacy Questionnaire was developed to evaluate postgraduate veterinary students' research competencies. The instrument consists of four dimensions:

1. Literature Retrieval Skills
2. Critical Appraisal Ability
3. Methodological Understanding
4. Scientific Writing and Research Design

Each dimension contains five items (20 items in total).
All items are rated using a 5-point Likert scale:
1 = Strongly disagree
2 = Disagree
3 = Neutral
4 = Agree
5 = Strongly agree

2. Scoring Method

- Dimension score = Sum of the five items within each dimension
- Total score = Sum of all 20 items
- Higher scores indicate higher perceived research literacy

Possible total score range: 20–100
The dimension scores reported in Table 2 correspond to the summed scores within each dimension.

3. Questionnaire Items

Dimension 1: Literature Retrieval Skills

1. I can effectively search major biomedical databases (e.g., PubMed, Web of Science).
2. I am able to construct appropriate search strategies using keywords and Boolean operators.
3. I can identify high-quality and relevant research articles.
4. I am proficient in using reference management software.
5. I can distinguish between systematic reviews and narrative reviews.

Dimension 2: Critical Appraisal Ability

6. I can evaluate the methodological quality of research studies.
7. I understand common sources of bias in scientific research.
8. I can correctly interpret forest plots.
9. I can understand and interpret heterogeneity statistics.
10. I can judge whether conclusions are supported by the data.

Dimension 3: Methodological Understanding

11. I understand the standard process of conducting a systematic review.

12. I can formulate appropriate inclusion and exclusion criteria.
13. I understand the difference between fixed-effects and random-effects models.
14. I am able to extract and organize data for analysis.
15. I understand the importance of reporting guidelines (e.g., PRISMA).

Dimension 4: Scientific Writing and Research Design

16. I can formulate a clear and focused research question.
17. I understand how to design a basic research protocol.
18. I can interpret basic statistical outputs.
19. I am able to write a structured scientific manuscript.
20. I understand ethical considerations in research reporting.

4. Administration

The questionnaire was administered before and after training in both groups. Responses were anonymized and used solely for educational evaluation purposes.
